# General practitioners referring patients to specialists in tertiary healthcare: a qualitative study

**DOI:** 10.1186/s12875-019-1053-1

**Published:** 2019-12-01

**Authors:** Konstantinos Tzartzas, Pierre-Nicolas Oberhauser, Régis Marion-Veyron, Céline Bourquin, Nicolas Senn, Friedrich Stiefel

**Affiliations:** 1 Center for Primary Care and Public Health (Unisanté), University of Lausanne, Rue du Bugnon 44, 1011 Lausanne, Switzerland; 2 Faculty of Social and Political Sciences, University of Lausanne, UNIL, Lausanne, Switzerland; 3 Psychiatry Liaison Service, University Hospital of Lausanne, Lausanne, Switzerland

**Keywords:** General practitioner, Referral process, Qualitative research, Primary care, Tertiary healthcare

## Abstract

**Background:**

There is a large and unexplained variation in referral rates to specialists by general practitioners, which calls for investigations regarding general practitioners’ perceptions and expectations during the referral process. Our objective was to describe the decision-making process underlying referral of patients to specialists by general practitioners working in a university outpatient primary care center.

**Methods:**

Two focus groups were conducted among general practitioners (10 residents and 8 chief residents) working in the Center for Primary Care and Public Health (Unisanté) of the University of Lausanne, in Switzerland. Focus group data were analyzed with thematic content analysis. A feedback group of general practitioners validated the results.

**Results:**

Participating general practitioners distinguished two kinds of situations regarding referral: a) “clear-cut situations”, in which the decision to refer or not seems obvious and b) “complex cases”, in which they hesitate to refer or not. Regarding the “complex cases”, they reported various types of concerns: a) about the treatment, b) about the patient and the doctor-patient relationship and c) about themselves. General practitioners evoked numerous reasons for referring, including non-medical factors such as influencing patients’ emotions, earning specialists’ esteem or sharing responsibility. They also explained that they seek validation by colleagues and postpone referral so as to relieve some of the decision-related distress.

**Conclusions:**

General practitioners’ referral of patients to specialists cannot be explained in biomedical terms only. It seems necessary to take into account the fact that referral is a sensitive topic for general practitioners, involving emotionally charged interactions and relationships with patients, colleagues, specialists and supervisors. The decision to refer or not is influenced by multiple contextual, personal and clinical factors that dynamically interact and shape the decision-making process.

## Background

General practitioners’ (GPs’) referral to specialists has legal and ethical dimensions, as inadequate referral can seriously undermine the quality of care [1–3]. However, GPs’ referral practices have yet not been comprehensively investigated, even if they are performed routinely. There is a significant and multifactorial variation in GPs’ referral rates to specialists. This variation remains largely unexplained, as less than half of it can be linked to patient, practice or GP factors [4–6]. Studies focusing on the referral process are therefore greatly needed, [5] notably qualitative studies providing new insights and hypotheses regarding how GPs are experiencing and conducting this process [3, 7]. Such studies seem especially important since there might be a gap between the lived reality of the referral process and its theoretical or administrative depictions, particularly with respect to the GPs’ concerns, feelings and attitudes [8].

These issues are especially important in the Swiss healthcare system. In Switzerland, health insurers reward patients for seeing a GP before consulting a specialist (37% of patients’ insurances) [9]. This context creates an equivalent to the gatekeeping system, [9] 67% of the population seeing a GP at least once a year (36% consult a specialist on their own initiative during the same period) [10]. One of Swiss patients’ main expectations towards GPs is an adequate coordination of care [11]. GPs’ essential role in the coordination of healthcare is widely proven, especially for the chronically and “complex” patients [1, 12]. On the contrary, inadequate referral can undermine the quality of care and lead to the misuse of resources [3, 13–16]. While Swiss GPs resolve 94.3% of all problems encountered, a specialist referral rate of 9.44% has recently been reported. This is three times as much as it was in 1989, but similar to rates measured elsewhere, notably in the USA [1, 17]. The “prescription” of a specialized intervention has thus become a daily activity of Swiss GPs.

The aim of this study is to contribute to a better understanding of the referral process by investigating what leads GPs to initiate or not a referral [18]. More precisely, we have tried to identify the factors that GPs working in a Swiss university clinic consider when pondering whether they should refer a patient to a specialist. Indeed, to the best of our knowledge, no qualitative inquiry has studied the referral process from the point of view of GPs, nor questioned their experiences and preoccupations related to referral.

## Methods

The study, conducted at the Center for General Medicine (CGM) of the University of Lausanne, in Switzerland, took place between December 2016 and June 2017, following approval by the Cantonal Ethics Committee for Research on Human Beings (CER-VD). The CGM is part of the Center for Primary Care and Public Health (Unisanté), proposing primary health care to Lausanne’s general population (a population of 400′000). Patients visit the CGM for any health-related problem, either after an appointment or as an emergency. CGM GPs offer first-line treatments and follow-ups. Located besides the Lausanne University Hospital, CGM directly collaborates with its specialists so as to provide coordinated outpatient primary care. It is also engaged in continuous collaborations with specialists in nearby private practices. The CGM is a referral center for internal and general medicine and the only university center training future GPs for the surrounding area. It is composed of 40 GPs (residents and chief residents). During 2017, CGM GPs conducted more than 18′000 consultations and followed up 4′000 patients. About 40% of consulting patients have psychosocial vulnerabilities [19]. These particular features should be kept in mind in order to avoid overgeneralizing of our results. Furthermore, the fact that most GPs participating in our study are young clinicians, usually still in training, can have an effect on the way they refer to specialists and experience the referral process.

The first step of our research plan was to ensure that our setting was appropriate for observing and investigating the referral process. We created a questionnaire on referral, based to the existing literature, and conducted a survey among CCM GPs in order to compare the existing literature findings with the studied population. Our survey’s results (*N* = 31) showed that in the CGM setting, the referral process is significantly important, being principally conducted by the residents. This makes the CGM a suitable setting for observing the referral process. The questionnaire’s results were used to develop the questions of the Focus Groups’ (FGs’) moderator’s guide (see Additional file 1).

A first FG was conducted with residents [20–22]. The last author (FS) with extensive experience in conducting FGs acted as moderator. To have a more complete view of the studied phenomenon, a FG was conducted with chief residents. The basic hypothesis underlying the choice to distinguish between residents and chief residents was that seniority, status, and decisional power could have an effect on referral and on GPs’ experience of the referral process (different roles, levels of responsibility within the CGM, clinical experiences, etc.) [23, 24].

The FGs were audio recorded and the main investigator transcribed them manually. The transcripts were analyzed by means of a qualitative approach. The two main investigators, a consultation-liaison psychiatrist (KT) and a social scientist (PNO), independently conducted a thematic content analysis on the transcribed FGs, with a specific focus on GPs’ self-reported concerns about the referral process and decision [22, 25, 26]. A deductive-inductive approach was used during coding. Based on the questionnaire’s results, the two main investigators agreed on an “a priori” analytical framework specifying key themes and questions. They then transformed this framework during analysis when it revealed inadequate to deal with the data [27]. The analysis was conducted independently by the two main investigators, which resulted in two slightly different sets of codes. They then confronted their findings and created an analytical model describing the main features of GPs’ decision-making process during referral.

At this point, the ongoing analysis was discussed with the other investigators. This discussion made clear that the two main investigators had been too focused on the decision-making process, setting apart other elements (“tactics” and referral “facilitators”, see below). The two main investigators reviewed again the transcripts independently, taking care to include these aspects they had previously left out. A dynamic model was developed, accounting for participating GPs’ distinction between what they see as “clear-cut” and “complex” situations and identifying stress-reducing “tactics” used by GPs as well as what they see as referral “facilitators”. The other investigators validated these results.

During the data processing stage, as well as during the data analysis and interpretation stage, the emerging themes were submitted to a feedback group of CGM-GPs. The developed “model” of the referral process was also submitted to this group. We went back and forth until we confirmed that our interpretations were going in the right direction. Triangulation of methods and respondents’ validation increased the validity of our study.

## Results

Ten residents participated in the first FG and eight chief residents to the second FG.

### “Clear-cut situations” versus “complex cases”

During the FGs, GPs distinguished two types of situations regarding the referral process: 1) “clear-cut situations” and 2) “complex cases”. They expressed the feeling that some cases need no further thoughts. Facing such “clear-cut situations”, GPs reported that they do not hesitate:

“There are situations in which it is very clear that we need a specialist. For example, we have a patient with typical, uh… chest pain. Or even atypical, but who has risk factors, so we say to ourselves that we can’t waste time and we must exclude the cardiac origin. So it seems pretty obvious that you need a stress test…”

Participating GPs said that these “clear-cut situations” are rare in their current working context, but occur much more often in private practices or in secondary care centers:

“When you're doing an internship in a GP's office… I was in the countryside… We saw many more patients a day than here, but then they were much ‘simpler’. […] The point is, referring or not is often clearer with ‘simple’ patients.”

Indeed, they believe that they encounter many “complex cases” at the CGM. “Complexity” here doesn’t mean that the intervention of a specialist is needed, but that it is difficult for GPs to decide if such intervention is necessary or whether it would be beneficial. When GPs are confronted with these situations, they often feel lost, not knowing how to proceed:

“We have complex patients who have a lot of comorbidities and treatments; and sometimes managing high blood pressure for example... One tells oneself: ‘But now I don't know what to do… Maybe I should have the specialists give me some advice.’”

Emotionally, “clear-cut” situations” and “complex cases” have contrasting significances. On the one hand, cases in which the decision to refer is difficult to make are linked by GPs to stress and anxiety. On the other hand, cases in which referring or not is “obvious” can give them the feeling of being “nothing more” than “sorter physicians”:

“[…] writing referral demands, eventually it becomes really frustrating and I think that it’s… If what’s expected of a GP is being a ‘sorter physician’, there won’t be many candidates for our profession…”

Such remarks reveal that referring is very significant for GPs with regard to how they perceive themselves, as opposed to specialists.

### Decision-making facing “complex cases”

Participating GPs reported that the decision to refer or not can be multi-layered, multifactorial and thus quite difficult to make in the “complex cases”. They attempt to continuously maintain a delicate balance between the “quality” and “safety” of care, taking into account the possible drawbacks of referrals:

“This is exactly the point regarding the notion of gatekeeping, which is really a delicate balance between quality and security of care. So if we are called to act as gatekeepers, to what extent are we supposed to do it or not?”

More importantly, our study reveals that GPs entertain diverse concerns regarding cases in which they hesitate to refer. These concerns can be classified into three different categories: a) concerns about the treatment, b) concerns about the patient and the doctor-patient relationship and c) concerns about the referring GP himself.

A) Regarding the concerns about the treatment**,** participating GPs indicated that they turn to specialists to optimize medical care when confronted with their own limits (theoretical, clinical or practical). In such cases, they refer to specialists for specific examinations or procedures they can’t or aren’t confident to do by themselves:

“Referring also brings security… Confidence… When we have some information, guidelines, but aren’t experts […]. Even when we go and look into the existing literature, we are never sure that we have the last guidelines…”

However, GPs also stated that they sometimes use referrals to delegate tasks to specialists in order to concentrate on other aspects of treatment. In such cases, they seem to use referrals in an “instrumental manner” to save consultation time that they want to use differently, creating a specific division of labor between themselves and the specialists:

“Once a plan of care has been established and some of the problems are handled [by the specialist], we can make time for more psychological, social, personal matters… Er… It’s a way to move forward…”

B) The second set of concerns regards the consequences of referring for the patient and for the doctor-patient relationship. GPs notably reported being quite preoccupied with the financial and/or psychological “cost” of referrals, especially in the case of the more vulnerable patients:

“For some of my patients, there has been no advantage at all [in the referral]. It was terribly stressful for them … Often, they don’t understand French and some specialists don’t ask for a translator to be present, even when we mention it on our request… They aren’t explained anything, and… They come back to us, and we have to explain what the specialist reported…”

Participating GPs said they therefore meet the explicit demands of patients with some caution, since seeing a specialist could influence the patient’s emotional state even positively or negatively, depending on the specialist-patient relationship:

"It depends on the contact they have with the specialist… There are patients who come back very upset because they have not been explained anything (M: Yes). While there... there are other times they come back with stars in their eyes, as if they had a revelation. […] That's right, it depends a lot on how consultation happens…"

Participating GPs – especially chief residents – also expressed concern about the possible effects of referrals on their relationship with the patient. On the one hand, they said they worry that patients would be disappointed if they didn’t agree to let them see a specialist. On the other hand, they evoked that they sometimes fear that referring could undermine the patient’s confidence in their judgement, or that meeting a specialist would prompt patients to compare their respective knowledge and skills:

“When we decide to refer or not, it is often difficult to know if we are doing too much or not enough. If we ask for advice all the time, the patient may feel insecure, because [he may think:] ‘Hell, this doctor is insecure!’ But if we decide not to refer, [he may think:] ‘This doctor does nothing but wait some more.”

Considering these various aspects, GPs expressed the feeling that referring means adding a “third party” to the “dyadic” doctor-patient relationship, which inevitably changes the relationship’s balance and dynamics. This fact is considered by GPs before referring:

"The issue of the relationship of course, we have… A dyadic relationship between physician and patient, which can be stable or not, but if we… We add a third contributor, the relationship won’t be dyadic anymore. So it’s very important to know why this third contributor is necessary. […] Because of course, if something goes wrong between the patient and the specialist, it will necessarily impact the relationship between the patient and the family physician.”

C) A third set of concerns regards the possible consequences of the referral for GPs themselves. Indeed, GPs mentioned the wish to share responsibility in order to be legally “covered” or to conform to institutional expectations:

“But we tell ourselves that we are still obliged to cover ourselves. If tomorrow a patient leaves and we miss something, it will be in the newspapers and then it can get bigger and bigger… If we make a mistake it's a bit of a disaster, and… Especially as we’re in an academic institution…”

These factors are evoked by GPs as prompting them to refer. However, they also express the fear that unnecessary or too numerous referrals might be taken as a sign of incompetence by specialists, patients, colleagues or supervisors. It seems important for GPs’ self-esteem to feel and demonstrate to others that they are able to manage things “on their own”:

“Or narcissism… I mean, yeah: ‘I can do it! Why wouldn’t I do by myself?’ […] ‘Yeah, I’ll read on the weekend, and I’ll do it.”

Viewed as such, abstaining from referring can be experienced as a “challenge” to be taken up, especially as CGM-GPs reported they feel some sort of latent competition with specialists.

### “Tactics”

Participating GPs also addressed the ways they try to relieve the decision-related distress experienced in “complex cases”. We describe such behavior as “tactics”, i.e. attempts to make a situation easier to face without radically altering it. They reported using two distinct “tactics” when hesitating to refer or not: a) *seeking advice of colleagues* (specialists or GPs) and b) *postponing the referral* and adopting a “watchful waiting” approach.

A) Regarding the first, GPs indicated that they often get advice before referring, either by soliciting the informal opinion of a specialist they personally know, or in a more formal way by requesting assistance from their supervisor. Such interactions help them to better comprehend the case at stake simply by describing it to someone else:

“I also often talk with my colleague from the next office because… If it’s a situation where I’m stuck a little bit, if I’m not very sure whether I should refer or not, it will give me the opportunity to summarize the situation orally to somebody. Well, sometimes it helps to see things more clearly…”

In addition, participating residents emphasized their supervisor’s influence on their decisions regarding referrals.

B) As to the second “tactic”, the use of “watchful waiting”, GPs indicated that they sometimes choose to postpone referring when they hesitate:

“And then there is the question of time, too. Can we wait a little longer before sending to the specialist? Try other treatments, err...”

Of course, GPs reported that they use such “tactic” only in “non-emergency” cases. Eventually, emerges the question of how long can the referral decision be postponed.

### Referral “facilitators”

Participating GPs mentioned various factors that facilitate the referral process, namely a) *internal training*, b) *guidelines*, and the c) *availability of colleagues, specialists and/or supervisors*.

A) GPs expressed that internal training has an important impact on their referring practices, as it can lead them to handle certain situations with much more confidence and/or without the help or advice of a specialist.

“We also have training. For example, lately there has been a symposium on gastroenterology about what to do in healthy adult in gastroenterology, so it allows us not to send everyone to gastroscopy… [M: Internal training] Yes, which is really suitable for generalists.”

B) GPs expressed the need to rely on clear theoretical backgrounds when referring, with guidelines being perceived as supporting and facilitating referral decisions. However, participating GPs’ attitudes towards guidelines are more ambiguous, as they consider guidelines as “forcing” some referrals that could have been avoided. In this perspective, guidelines appear as an institutional pressure rather than as a decision support tool:

“And then there is the question of guidelines. Sometimes we are quite sure about the psychosomatic origin [of the patient’s symptoms], but we say to ourselves: ‘theoretically, we should nevertheless send him to a specialist’…”

C) Finally, chief residents evoked the availability of specialists and the quality of their relationship with them as important for the referral process. They regretted not benefiting enough from more proximity with the specialists:

“[…] with a network of specialists, knowing each other, knowing the specialists we work with. We would have a contact that would be different, it would be easier to ask for advice.”

They underlined that personal relationships with specialists facilitate the referral process and can help them in their decision-making.

## Discussion

### Summary

The aim of this study was to contribute to a more accurate understanding of how GPs decide to refer their patients to specialists [3, 6, 7, 18]. To this end, we analyzed two FGs conducted among GPs (residents and chief residents) working in a university outpatient clinic, located besides the Lausanne University Hospital. Most GPs participating in our study were young clinicians, more than half of them still in residency training. An important number of patients visiting the clinic have psychosocial vulnerabilities. These are the specific features of our study’s setting. When asked about what comes into play during the referral decision, participating GPs distinguished between “clear-cut situations” and “complex cases”. They think that “clear-cut situations” are less common in their working context compared to other healthcare settings. Nevertheless, they believe that internal guidelines and training help them to feel more confident when deciding to refer or not.

Regarding the “complex cases” in which the decision to refer or not is more difficult to make, GPs reported various concerns: a) about the treatment, b) about the patient and the doctor-patient relationship and c) about themselves. The first set of concerns address the issue of adequate treatment and optimal coordination of care. The decision to refer is mainly motivated by the notion that the specialist knows and/or can do more for the problem at stake. GPs also reported that they sometimes use referrals in an instrumental way, in order to gain time and room for focusing on other aspects of the patient. Regarding the possible consequences of referral for the patient and for the doctor-patient relationship, GPs showed concern over the financial and/or psychological “cost” for patients. They also expressed ambivalent feelings concerning the possible effects of referrals on the doctor-patient relationship, with the specialist intruding as a “third party” in their “dyadic” relationship with the patient. Finally, participating GPs underlined that they are at times concerned for themselves and associate referral with the desire to be legally “covered” or to meet institutional expectations and with the fear of specialists’, patients’, colleagues and/or supervisors’ judgments regarding their decisions.

Participating GPs reported that they attenuate the decision-related distress linked to some of the “complex cases” by a) asking colleagues (specialists or GPs) or supervisors for advice and b) postponing the referral (temporizing). The main contextual factors influencing the referral process were a) internal training, b) guidelines, and c) access to colleagues, specialists and/or supervisors.

**Fig. 1:**
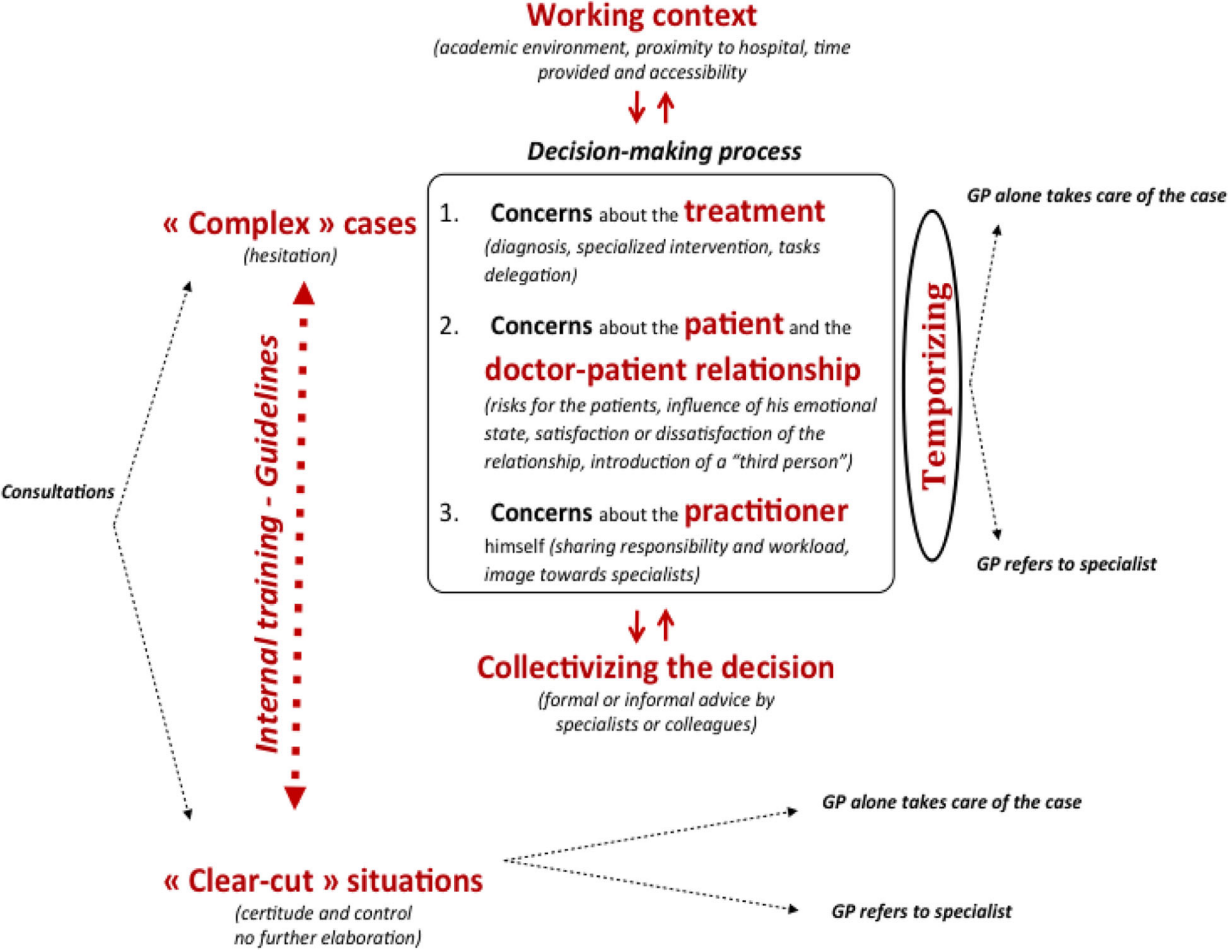
The Referral Process as experienced by general practitioners

Figure 1 below summarizes these findings. It should not be understood as an “objective” depiction of the referral process, but as a representation of GPs’ lived experience of the referral process (Fig. 1).

### Strengths and limitations

Although the need for qualitative studies addressing the referral process has been acknowledged, [3, 7, 14] little research has been conducted so far. Our study contributes to the effort to approach qualitatively such a phenomenon, by investigating CGM GPs’ expectations, thoughts, feelings and concerns when referring patients to specialists. The study thus allowed to shed light on what clinicians experience and take into account when deciding whether they should refer patients to specialists or not.

Nevertheless, there are three obvious limitations to our study. First, the perspective we have chosen is general: we did not address one specific type of referral, since referrals to various specialties (e.g. psychiatry, cardiology, orthopedics, etc.) may present specific challenges. However, we feel our choice was warranted for at least two reasons: a) we wished to identify some general, basic assumptions about the referral process, [8] especially in its theoretical depictions, which presume that the factors affecting the referral decision are purely biomedical; b) participating GPs themselves seemed to confirm that “referral” could be addressed as a unitary category. Second, our purpose being to document GPs’ points of view and experiences about the referral process, we did not interview patients, specialists, or supervisors. Their inclusion would certainly add to the understanding of the referral process.

A third limitation of this study results from the specificity of the setting. However, the particularities of our study’s setting are consistent with the aim of our study, and correspond to a typical setting in which multiple reasons for referral exist: the CGM is a university outpatient primary care clinic contiguous with a university hospital, in which GPs are confronted to complex clinical cases and work in constant collaboration with different specialists. In addition, the CGM treats patients with psychosocial vulnerabilities, in need of multidisciplinary care, who are usually emotionally challenging for GPs [1, 12, 28]. In such a setting, referring to specialists is a central act in providing medical care and strongly preoccupies GPs, a condition that was seen as an advantage for investigating the referral process. In addition, the majority of participating GPs were in training or at an early stage of their career. We can hypothesize that young GPs with less clinical experience are more preoccupied about how to refer or not to specialists, and that therefore also various physician-related reasons for referral were prevalent. Accordingly, our setting was a very fertile ground for studying the referral process.

### Comparison with existing literature

The problems linked to the referral process call for models which optimize care by facilitating “adjustment” of referral attitudes between GPs and specialists [1]. Such models should be based on healthcare workers’ expectations, experiences and affects and on qualitative studies that offer a better understanding of referral [3, 6, 12, 18, 29–31]. By focusing on GPs lived experience, our study contributes to this effort. It provides a new perspective on the referral process and the associated decision making process, as most researchers have so far addressed this issue by solely scrutinizing the biomedical factors that influence referral.

The main themes tackled by prior studies focusing on referral are: a) GPs’ need for a better access to specialists [12, 32]; b) importance of suitable communication and good relationships between GPs and specialists [2, 4, 12, 15, 32–35]; c) effects of referral on the doctor-patient relationship [2, 30, 32, 36, 37]; d) referral and heavy workload (resistance, transfer of responsibility, lack of specific training on how to “prioritize”) [2, 24, 30, 32, 37]; e) uncertainty linked to referral [4, 5, 24, 30]. These themes match our own results. Participating GPs evoked that they often struggle to decide whether or not they should refer patients to specialists, and that they ask colleagues for advice and/or postpone their decision. Furthermore, they expressed the wish for a better access to and relationship with specialists and presented specialists’ availability as facilitating element of the referral process. They also explained that they sometimes see referral as a way to share responsibility with specialists in order to be legally “covered”, to delegate tasks to specialists and to take advantage of their specific knowledge. The influence of referrals on the doctor-patient relationship has also been widely reported by CGM GPs in the FGs.

However, some aspects discussed in other studies don’t appear in our results, such as the “unrealistic” character of GPs’ expectations [13, 14] and their feelings of inferiority towards specialists [38, 39]. This is hardly surprising, as these elements tend to negatively depict GPs involvement in the referral process. In a similar way, it is worth noting that participating GPs didn’t explicitly call for an improvement of primary healthcare structures or for a more intensive patients’ involvement in the referral process, as described in the literature [1, 3, 30].

Globally, our research replicates the results of prior studies, but takes them a step further by expanding our knowledge of GPs’ experience of the referral process. Aspects that haven’t been described by previous studies include: the influence of patients’ emotions and specialists’ esteem towards GPs; GPs’ fears surrounding the issue of responsibility; the use of referral as a way to learn from specialists; and the desire for more training, guidelines and colleagues’ support regarding the referral process. Indeed, referral appears to be a central issue for GPs, which can produce various and sometimes strong emotional states. By investigating GP’s point of view of how interactions with patients, specialists and supervisors influence referring, we offer a deeper knowledge of the central issues surrounding this “prescription” [38]. Finally yet importantly, we also document some GPs’ self-reported “tactics” when facing complex referral situations.

### Implications for research and practice

Shedding light on the referral process is useful: a) for GPs, b) for healthcare systems planners and c) for university medical trainers. Being aware of thoughts, experiences and feelings associated with the referral process increases GPs’ optimal utilization of specialized care and positively influences the risk-benefit balance of referral [12, 18, 40, 41]. Strategies for reducing medical over−/underuse include GPs adopting a well-founded “wait-and-see” approach, [30] a better management of uncertainty [4, 5, 24] and a capacity of mobilizing formal or informal information in their working environment [7, 29]. The issues surrounding the referral process must be taken seriously into account by healthcare system planners, notably with regard to effective communication and coordination between participants and the creation of efficient healthcare networks [1, 3, 7, 12, 29, 42]. Finally, university GPs expect from their supervisors that they establish specific training and internal guidelines for referral, adapted to their specific working context [1, 29]. Therefore, medical trainers have to be aware of the multilayered interactions involved in the referral process [1, 3, 5]. Their clinical teaching should thus also focus on relationships and interactions in the referral process: a) the doctor-patient relationship; b) the GP-specialists relationship and c) the GP-institution relationship [38, 42–44].

## Conclusion

We have tried to identify more accurately the various grounds on which GPs decide to refer their patients to specialists, which is a central issue not only for GPs, but also for their patients, trainers, supervisors and healthcare planners [3, 6, 12, 29]. Our study reveals that various factors are associated with referral. There are certainly biomedical elements influencing the referral process, but most elements are associated with the GPs’ lived experiences, such as his own concerns, expectations and emotions or the perception of the patients’ psychological needs, and contextual factors, [16] such as training opportunities to address the referral process. It seems especially important to take into account the observation that referral can be a stressful experience for the practitioner himself, challenging his self-esteem and involving issues of recognition [4, 38, 45]. Since referral is a cornerstone of interactions among GPs, their patients, specialists and their supervisors, its optimal management is crucial [1, 11, 45, 46]. The various themes emerging from our research and the proposed conceptual model that organizes them contributes to a more comprehensive understanding of the referral process.

## Supplementary information

**Additional file 1.** Focus Group - Moderator’s guide

## Data Availability

The raw data supporting our findings is available from the Dryad Digital Repository and can be found in https://datadryad.org/stash/share/762cHyghHxTUTTKFeVxPLMbdLipYbB4JXz-9wYPjFS0

## Abbreviations

CER-VD: Cantonal Committee on Ethics for Research on Human Beings
CGM: Center for General Medicine
FGs: Focus Groups
FLHR: Federal Law on Human Research
GPs: General practitioners
