# Supplementary material for: General practitioners referring patients to specialists in tertiary healthcare: a qualitative study
Source: BMC Fam Pract. 2019 Dec 1;20:165. doi: 10.1186/s12875-019-1053-1 (PMC6885318; doi:10.1186/s12875-019-1053-1)
Supplement: Supplementary file 1 — Additional file 1. Focus Group - Moderator’s guide [file 12875_2019_1053_MOESM1_ESM.pdf]

Annex 1

<u>Focus Group - Moderator's guide</u>

"The three steps of the referral process"

1) When you are wondering if you should refer to a specialist:

- What motivates your decision to do so? (internal motivations, related to the patient, the specialist, the institutional context, current medical practice and expectations)
- What, on the contrary, makes you hesitate or give up calling on a specialist?
- Before you refer, why do you first discuss the situation with a colleague GP (senior practitioner or resident)?
- We note that the reference rate in the CGM is higher than the Swiss average (25% in the CGM >5% in Switzerland). How do you explain that?

2) When making the referral:

- What helps you make effective and appropriate use of specialists and how your training helps you in this?
- You can use indirect channels (e-mail, good grid, etc.) or direct (oral) - it appears from the survey that you prefer indirect contact. How do you explain that?
- In your opinion, what are the advantages/disadvantages of proceeding directly or indirectly?

3) Gains brought by the specialist

- What does the specialist's opinion ultimately bring to you, on the one hand, and what does it bring to the patient, on the other?
- Are there any disadvantages to calling in a specialist, again for you and for the patient?
